# Preventing domestic abuse for children and young people: A review of school-based interventions

**DOI:** 10.1016/j.childyouth.2015.10.018

**Published:** 2015-12

**Authors:** Nicky Stanley, Jane Ellis, Nicola Farrelly, Sandra Hollinghurst, Soo Downe

**Affiliations:** a School of Social Work, University of Central Lancashire, Preston, UK; b School of Social and Community Medicine, University of Bristol, Bristol, UK; c School of Health, University of Central Lancashire, Preston, UK

**Keywords:** Domestic abuse, Intimate partner violence and abuse (IPVA), Prevention, Dating violence

## Abstract

Schools provide the setting in which interventions aimed at preventing intimate partner violence and abuse (IPVA) are delivered to young people in the general population and a range of programmes have been designed and evaluated. To date, most rigorous studies have been undertaken in North America and the extent to which programmes are transferable to other settings and cultures is uncertain. This paper reports on a mixed methods review, aimed at informing UK practise and policy, which included a systematic review of the international literature, a review of the UK grey literature and consultation with young people as well as experts to address the question of what works for whom in what circumstances.

The context in which an intervention was delivered was found to be crucial. Context included: the wider policy setting; the national or regional level, where the local culture shaped understandings of IPVA, and the readiness of an individual school. The programmes included in the systematic review provided stronger evidence for changing knowledge and attitudes than for behavioural change and those young people who were at higher risk at baseline may have exerted a strong influence on study outcomes. Shifting social norms in the peer group emerged as a key mechanism of change and the young people consulted emphasised the importance of authenticity which could be achieved through the use of drama and which required those delivering programmes to have relevant expertise. While the consultation identified increasing interest in targeting interventions on boys, there was an identified lack of materials designed for minority groups of young people, especially Lesbian, Gay, Bisexual and Transgender young people. Increased responsivity to the local context can be achieved by involving those who will deliver and receive these preventive programmes in their development. Schools need to be better prepared and supported in the task of delivering these interventions and this is particularly relevant for the management of disclosures of IPVA. Outcomes measured by evaluations should include those relevant to education.

## Highlights

• National policy, culture and school readiness form a vital context in IPVA prevention.
• Programmes transported across cultures should incorporate local values and content.
• Authenticity emerged as a key ingredient of successful preventive interventions.
• Those children and young people experiencing IPVA may require additional support.

## 1 Introduction

The widespread nature of domestic abuse requires a multi-level response in which preventive interventions that target whole populations form a wide and substantial base to a pyramid of service responses. Schools provide a context in which such initiatives can be delivered on a large scale to a relatively captive audience who have yet to experience or are just embarking on their own intimate relationships. Since intimate partner violence and abuse (IPVA) in young people's relationship impacts on their immediate health and wellbeing (Barter, McCarry, Berridge, & Evans, 2009) as well as acting as a precursor for IPVA in adult relationships (Black et al., 2011) the gains may be short-term as well as long-term. Moreover, since much of children's social learning takes place in school, educational settings appear to offer an appropriate environment for delivering learning about domestic abuse (Sudermann, Jaffe, & Hastings, 1995). Such thinking has resulted in the development of a range of preventive domestic abuse programmes designed to be delivered in schools; in North America, these are usually described as dating violence programmes while in the UK, where ‘dating’ is not a term commonly used by young people, they go under the label of healthy relationship programmes or domestic abuse or awareness raising programmes.

Domestic abuse, as it is usually termed in the UK (in this paper, we use the terms domestic abuse and IPVA interchangeably), has been described as a ‘wicked problem’ (Devaney & Spratt, 2009) meaning that its complexity requires a multifaceted response which may be partial in its success. Gender inequality is usually identified as a structural factor underpinning domestic abuse but Harvey, Garcia-Moreno, and Butchart's (2007) WHO paper on primary prevention identifies eight risk factors for IPVA and sexual violence which include poverty, gender inequality, a lack of support from criminal justice services, weak community sanctions, dysfunctional relationships, substance misuse, childhood experience of violence and social norms that support traditional gender roles and IPVA. While programmes delivered in schools are only one approach to prevention in this field, they are arguably the most widely tested approach and they have been ‘scaled up’ with widespread implementation of some programmes in the USA, Canada and Australia (Lundgren & Armin, 2015).

However, to date, the evidence for the effectiveness of such programmes has been judged to be limited (Fellmeth, Heffernan, Nurse, Habibula, & Sethi, 2013) and as much of the evidence base has been generated in North America, there are questions about its transferability (Flood, 2015; World Health Organisation (WHO)/London School of Hygiene and Tropical Medicine, 2010). The mixed methods review reported here sought to move beyond simple measures of effectiveness to consider what works for whom in what circumstances and to explain the process of change (Pawson, Greenhalgh, Harvey, & Walshe, 2005).

## 2 Background to the study

This review focused on the UK context where these preventive programmes have been delivered for a period of about 25 years (Ellis, Stanley, & Bell, 2006). Despite this established history of provision, the availability of such interventions is known to be variable and ad-hoc, with much of the development and implementation of programmes undertaken by the independent sector where funding is often limited and short-term (Stanley, Ellis, & Bell, 2010). The delivery of programmes in schools is often determined by the enthusiasm of one individual and it is rare for children to receive regular exposure to domestic abuse prevention initiatives across their school careers. The UK policy picture is similarly variable. Although the definition of domestic abuse has been extended in England and Wales to include IPVA experienced by young people aged 16–17 years of age (Home Office, 2013), preventive education on IPVA is not a mandatory part of the curriculum in England. In contrast, in Northern Ireland and Scotland, preventive education on IPVA is delivered on a mandatory basis while the Welsh Government has announced plans for this to happen.

The UK research landscape reflects the patchy picture of policy and practise in that there are no UK based trials and much of the research to date has taken the form of local before and after studies, often with integrated process evaluations. Some of these studies are only available as grey literature, that is, publications which are not produced through normal commercial publication channels (Auger, 1994). This review therefore aimed to include a wider range of evidence than previous systematic reviews of school based programmes in this field, two of which are restricted to consideration of randomised or quasi-randomised trials (De Koker et al., 2014, Fellmeth et al., 2013). Fellmeth et al.'s (2013) meta-analysis included interventions for young adults as well as children and the authors found no significant effects for all outcomes with the exception of knowledge change. They concluded that the lack of evidence for effect indicated the need for further and more rigorous studies. De Koker et al. (2014) reviewed eight papers and one trial report which together reported on six RCTs of preventive IPVA interventions for young people aged 11–26. They found more evidence of effectiveness for those four programmes that incorporated a community based component and reached the cautious conclusion that multi-component interventions are more effective. These reviews mainly focus on outcomes in respect of behavioural change, specifically perpetration of intimate partner violence and victimisation. Whitaker et al.'s (2006) review included a wider scope of material, addressed a broader range of outcomes and was more optimistic in its conclusions, finding that nine of the studies reviewed reported at least one positive outcome relating to either knowledge or attitudes. However, it only included material published up to 2003 so there is no current systematic review of non-randomised evaluations available that includes data from studies undertaken over the last 12 years.

## 3 Review methods

This mixed methods review (Gough, Oliver, & Thomas, 2012) aimed to capture the complexity of these preventive interventions by drawing on a variety of sources and engaging a wide range of stakeholders in the study. Informed by Realist review principles that emphasise the relevance of stakeholder priorities, the significance of theories that inform interventions and the processes that might explain programme effects (Pawson et al., 2005), it comprised four elements: a systematic review of the international published literature together with a review of the UK grey literature; consultation with stakeholders including young people, experts from education and from research policy and practise in domestic abuse as well as a mapping survey and analysis of data on programme costs and benefits. Findings from the mapping survey and cost benefit analysis are reported elsewhere (Stanley et al., 2015); here we concentrate on the findings from the literature review and the consultation undertaken as part of the study that addressed interventions delivered in schools.

The systematic literature review included studies reporting preventive interventions in domestic abuse for children and young people under 18 in all languages published between 1990 and 2014. The search strategy was deliberately wide and we chose not to restrict the review to RCTs in order to be able to include studies using a range of methods. The inclusion and exclusion criteria used are shown in Appendix 1. The databases searched comprised Allied and Complementary Medicine Database (AMED); Applied Social Sciences Index and Abstracts (ASSIA); Cumulative Index to Nursing and Allied Health Literature (CINAHL); EMBASE; Education Resources Information Centre; MEDLINE®; PsycARTICLES®; PsycINFO®; Social Policy and Practice; Social Work Abstracts; Sociological Abstracts; Studies on Women and Gender Abstracts; Australian Education Index; British Education Index and the Centre for Reviews and Dissemination NHS Economic Evaluation Database (NHS EED). These were searched electronically using search terms structured in accordance with the PICO (population, intervention, context, outcome) Framework. A summary version of the search terms used is provided in Appendix 2. Searches were undertaken first in 2013 and then updated in February 2014. In total, 82 papers were identified for full text screening and these yielded 28 quantitative papers covering 20 separate programmes and six qualitative studies reporting young people's views of programmes for the review. Three of the qualitative studies were included in the quantitative papers reviewed; one reported on the implementation of a programme also included in the quantitative review while two addressed different programmes so 22 programmes were included in the systematic review. Table 1, Table 2 identify these studies and summarise their key characteristics. A framework for data extraction was developed using the following headings: context, programme theory; mechanism including delivery and content, audience and outcomes. The characteristics of each study were also logged along with their quality scores. Quantitative findings were summarised narratively under four headings: measures of knowledge; attitudes and/or behaviours (such as help-seeking) as well as incidences of victimisation or abuse related to relationships. Separate analyses were done by gender; grade; age; and history of perpetration or victimisation at baseline. Qualitative data were analysed thematically using a modification of the meta-ethnographic approach (Noblit & Hare, 1988).

The review of the UK grey literature utilised the same time-frame as the systematic review and was planned to include local independent evaluations, national reports, technical reports and theses; in-house evaluations were excluded. These publications were identified from the systematic review, from a search of relevant websites, by backchaining and through requests to experts involved in the consultation process (see below). In total, 46 documents published between 1990 and March 2014 were identified and 18 independently conducted evaluations of programmes were reviewed. Data were extracted using the same approach as was employed for the review of published literature.

The consultation took two forms: nine meetings were held over a period of 18 months with three different groups, each meeting on three occasions. The first was an already constituted young people's group: a youth council that had experience of being consulted on similar social issues and had addressed domestic abuse along numerous other education and welfare issues in the past. The group was not designed to be representative but rather was a means of ensuring that young people's perspectives informed the review in the same way as did those of other expert groups. The membership of the group fluctuated between meetings: eighteen young people aged 15–19 attended the first meeting of this group with seven or eight young people attending subsequent meetings. The two other groups comprised professionals from education who met as one group while the other group included practitioners and policy makers involved in communication and campaigning on domestic abuse or young people's health and wellbeing. Recruitment to both these groups was informed by discussion with relevant education and domestic abuse organisations and aimed to achieve a blend of policy officers, practitioners and researchers in each group as well as ensuring representation from all four countries of the UK. The consultation groups took place in parallel with other elements of the review so it was possible to adopt an iterative approach whereby group discussion was stimulated by feedback from the study that included progress reports and early findings while themes from the consultation groups also fed into the design of research tools and into analysis and interpretation of the results.

The second approach to consultation involved 16 individual telephone interviews with international experts involved in the design, delivery and evaluation of preventive interventions in the US, Canada, Australia, New Zealand and the UK. Again, individuals were selected for interview through a process of consultation with relevant organisations and with members of the expert consultation groups. Only one expert approached by the researchers declined to be interviewed; another four did not respond to email requests. All groups and interviews were recorded and transcribed with the participants' permission. The involvement of the young people's consultation group was approved by the University of Central Lancashire's Ethics Committee.

Transcripts were analysed thematically by the lead author and checked by a second research team member using the main headings adopted for data extraction in the systematic literature review. In line with modified grounded theory approaches (Charmaz, 2000) sub-themes and new themes arising from the data were added as they emerged. The software package NVivo was used to assist with the sorting and storing of data. The findings from the different elements of the review were synthesised under headings corresponding to the framework for analysis used across the study: context; programme outcomes; audiences and processes. This approach is consistent with the Realist approach (Pawson et al., 2005) to reviews in which all data are synthesised to illuminate what works for whom under what circumstances. These headings are used below to structure reporting of the results.

## 4 Results

### 4.1 Context

Both the literature reviews and the consultations identified the context in which these preventive programmes are developed and delivered as crucial. At the *macro* level of national policy and guidance, the experts consulted argued that framing the delivery of preventive interventions in domestic abuse as a statutory requirement made for more consistent implementation as well as contributing to the climate in which social norms are created. Australia was cited as an example where national policy directives accompanied by central government funding for implementation of preventive programmes had been effective in embedding preventive domestic abuse programmes.

At the *meso* level of implementation in the region, a number of the North American programmes included in the systematic review that were judged to have been more rigorously tested had been developed in particular regions of the US with some, for example, Foshee et al., 1998, Foshee et al., 2000, Foshee et al., 2004, Foshee et al., 2005 Safe Dates Programme in rural Carolina or Taylor, Stein, Mumford, and Woods's (2013) New York evaluation of the Shifting Boundaries programme, trialled in predominantly rural or urban settings. The difficulties of transferring programmes across cultures and populations were apparent from the systematic review. Delivering the US developed programme, Coaching Boys Into Men, in India entailed substantial amounts of additional training for the facilitators who lacked the necessary awareness and attitudes required for delivery (Miller et al., 2014), while implementing Safe Dates in Switzerland (Hamby, Nix, De Puy, & Monnier, 2012) required considerable attention to be paid to language and cultural constructions of abuse. The need for such modifications suggests that programme fidelity may not always be an appropriate goal since conceptions of domestic abuse are culturally shaped and levels of gender equality and awareness of gender abuse differ between communities and societies. Home-grown and culturally specific interventions developed with input from those who will deliver and receive them may be most acceptable for those delivering programmes and more meaningful for the audience. The systematic review identified some US examples of programme designed for specific cultural groups (Belknap et al., 2013, Jaycox et al., 2006).

At the *micro level* of the school, organisational readiness to introduce a preventive intervention was identified as important by the experts interviewed. The consultation groups emphasised the need for interventions to be supported across all aspects of a school's work and curriculum, by the governors and senior management as well as through links with parents, the local community and relevant local agencies:

*You've got to have that whole school approach but then take it even further and the parents have got to be informed, the parents have got to be supporting the aims….*

(Education Consultation Group 2)

The review of the UK grey literature identified a small number of examples of the ‘whole school approach’ delivered in the UK. This approach is based on an ecological model where learning in the classroom is reinforced across the curriculum and in other aspects of school life. However, the evidence base to support such approaches is yet to be developed although an independent evaluation is available (Maxwell, Chase, Warwick, Aggleton, & Wharf, 2010).

In addition to the broad ‘whole school’ approach, members of the education consultation group also advocated a ‘spiral’ approach which extended across time and throughout a child's educational career so that learning about relationships and domestic abuse was reinforced by different parts of the curriculum at different times:

*We go through from children's centres to infant, nursery, to junior, primary, secondary, and obviously it's a dramatic change from children's centres to Year 13 in secondary school but…it's all cumulative.*

(Education Consultation Group 1)

### 4.2 Programme outcomes

Programme effectiveness has to be judged in the light of the outcomes selected for measurement. Table 1, Table 2 show that in most studies reviewed these were identified as changes in young people's knowledge, attitudes, behaviours, as well as incidence of victimisation or perpetration. Even where statistically significant findings were reported, the effect sizes were generally very low or, at best, moderate. The largest effect sizes were found in measures of knowledge, although the differences in these tended to decrease over time. The only relatively large and statistically significant finding in a well-designed study in terms of incidence of perpetration or victimisation, was found in Wolfe et al.’s (2009) evaluation of the Fourth R programme where perpetration of physical dating violence by boys participating in the programme was found to have decreased 2.5 years after the programme. However, it is worth noting that the much higher rate of reported perpetration of physical dating violence by girls when compared to boys in both intervention and control groups at both time points in this study was highly unusual.

Apart from this finding, the controlled studies included in the review found little differences in outcomes by gender. In contrast, 11 of the 12 case–control and cohort studies that looked for them found gender differences, although only a few were significant. Most of the differences showed better outcomes for girls. In respect of other variables, the systematic review found no strong evidence of effect across programmes and outcomes for ethnicity, age grade, level of English, or academic achievement.

Most of the programmes evaluated aimed to improve knowledge and awareness rather than achieving behavioural change. Increased knowledge and awareness have been identified as key to recognising domestic abuse in one's own or others' relationships and to help-seeking specifically (Humphreys and Thiara, 2003, Thomson et al., 2013). Most interventions in respect of abusive behaviour are based on the premise that behaviour only changes over time. This review did show that interventions based on information could increase knowledge in the short term. However, the retention of this knowledge in the longer term is less evident. An increase in help-seeking was found in some studies by both the quantitative and qualitative reviews.

A distinct skew in the data was found in a number of the studies included in the systematic review. Some authors were explicit in noting differences in characteristics of their sample which distinguished some groups as being at higher risk at baseline (Foshee et al., 1998, Foshee et al., 2004, Lavoie et al., 1995, Pacifici et al., 2001), while other studies were found to have skews in the characteristics of their intervention and control groups at baseline and/or follow up but there was no comment included as to whether this had influenced outcomes (Avery-Leaf et al., 1997, Black et al., 2012, Hilton et al., 1998, Macgowan, 1997, Miller et al., 2012, Miller et al., 2014, Taylor et al., 2013, Weisz and Black, 2001, Wolfe et al., 2009). Young people who were at higher risk at baseline may have exerted a strong influence on study outcomes and this indicates that programmes may be more or less effective for certain sub-groups, depending on how far these influences are identified and taken into account. One task for these programmes is to identify those who have already been exposed to IPVA either in their own or their parents' relationships and offer relevant support. This issue is discussed further below.

### 4.3 Audiences

While the systematic review found no programmes that reported outcomes for children under ten, the review of the UK grey literature included four programmes for primary school children under 11 years; six programmes aimed at school children of all ages and two programmes designed to be delivered to children of all ages in young people's centres outside school. Those programmes delivered to children under 10 were less likely to address domestic abuse directly but rather focused on wider relationship issues such as friendship, respect and children's safety. Domestic abuse was more likely to be explicitly identified in programmes for children aged eight years and over. Those programmes that were designed for both under 10s and adolescents entailed two separate but complimentary programmes (Ellis, 2006, Hale et al., 2012, Reid Howie Associates, 2002) which notionally offered opportunities for progression and continuity.

As noted above, the systematic review produced mixed findings concerning the relationship between gender and outcomes and Wolfe et al.’s (2009) evaluation of the Fourth R programme was the only study to show better outcomes for boys. The expert consultation groups and interviews revealed that boys were increasingly identified as a primary target for change and it was argued that this was a more effective strategy than encouraging girls to recognise and avoid victimhood:

*If you are aiming these programmes that are trying to somehow help girls be victimised less then it's tough because really it's totally up to whoever might victimise them to change their behaviour…Primarily, you want to target potential perpetrators….*

(Expert 1, USA)

It was generally agreed across all forms of consultation that messages for boys should be positively framed and should avoid a blaming approach that could provoke resistance. The qualitative literature included in the systematic review (Bell and Stanley, 2006, Fox et al., 2014) reviewed yielded examples of some boys who reported finding the programmes ‘anti-men’ or ‘sexist’ and resisted programme messages.

However, with the exception of interventions for boys, it was a consistent finding across all elements of this study that interventions rarely took account of diversity within the population of children and young people. Whilst data from the systematic review and expert interviews showed that in North America, Australia and New Zealand a small number of programmes paid attention to addressing the complexities of domestic abuse for children and young people marginalised through race/ethnicity, class, sexuality or disability, there was little evidence of such interventions being widely developed in the UK context. The consultation groups identified a need for programmes that were tailored to the needs of disabled children, including children with autism, and children from Black and Minority Ethnic groups. The lack of materials designed for Lesbian, Gay, Bisexual and Transgender (LGBT) young people was repeatedly emphasised:

*… [LGBT] young people we spoke to definitely didn't think they were addressed at all. They just felt pushed aside and isolated by discussion of relationship abuse or sex education.*

(Education Consultation Group 1)

### 4.4 Processes

#### 4.4.1 Peer group power

Most of the programmes included in the systematic review were underpinned by an explanation of domestic abuse that drew on social norms and feminist or gender theories and those interventions utilising the ‘bystander approach’ (Katz et al., 2011, Miller et al., 2012), which encourages young people to intervene and challenge abusive behaviour and language when they encounter them, made explicit use of peer group attitudes and behaviour as a mechanism of change. Similarly, those involved in the expert groups talked about shifting the climate or ‘creating conversations’ as an aim of preventive interventions. Interventions aimed at adolescents in particular have the opportunity to harness peer group values and attitudes to the task of changing behaviour and most young people hold positive attitudes in relation to violence and abuse (Burman & Cartmel, 2005) which programmes can articulate and strengthen. Delivering these programmes in a group setting provides opportunities to use the power of the peer group to construct social norms that challenge domestic abuse and this use of an informed and aware peer group was discussed by those involved in the consultation groups and interviews:

*…in any classroom of 25 kids, five of those kids might be at risk, five or even ten of them might be at risk of an abusive relationship. The other 15 are there to keep that from happening… the other kids know what to say, the other kids they now have the language, so that peer component is critical.*

(Expert 2, Canada)

#### 4.4.2 Authenticity

Authenticity was a key ingredient of successful interventions identified by those involved in the consultations, particularly young people themselves. Authenticity had a number of dimensions. It could be achieved through the use of messages and material that were recognisable and meaningful to young people and which made ‘*it real’*. For instance, in discussing the Home Office's *This is Abuse* television campaign, which featured young people close in age to them, members of the consultation group commented: *‘...because of our like age group, we could relate to it a bit more, it seems more real’*. (Young People's Consultation Group 2).

Authenticity was also enhanced when interventions were delivered by those with relevant expertise or experience and the young people consulted contrasted the genuine nature of such messages with those that were delivered by individuals who lacked conviction or plausibility.

Drama, theatre, real life accounts and narrative are often components of these preventive interventions and both the young people and experts consulted argued that such approaches had the potential to deliver an emotional charge which contributed to authenticity and promoted imaginative identification:

*It's like in front of you and then you realise, actually, it doesn't happen miles away, you know, it happens here. And it's so close to home and it happens to people that you might know and, you know, it can easily happen to anyone. And so I think drama kind of conveys that a bit more.*

(YP Consultation Group 3)

The young people consulted were conscious that not all young people had the confidence to participate in drama and they argued that both participative drama and non-participative theatre could succeed in engaging young audiences.

#### 4.4.3 Who delivers?

The review identified debate in the literature concerning which professional group should deliver programmes in school. While many of these programmes have been developed in the independent domestic abuse sector and reflect the gendered perspective and understanding of domestic abuse of that group of practitioners, teachers were identified as owning the relevant teaching skills, being better placed to both embed programmes in the curriculum and to follow up on any issues raised for children subsequent to delivery of a programme (Fox et al., 2014). However, the UK grey literature included examples of teachers who lacked the confidence and values required to deliver these programmes and members of the education consultation group noted that teachers were often ‘outside their comfort zone’ with this form of education. Young people participating in the consultation noted that teachers' lack of assurance and expertise could undermine programme messages:

*If it's like just a teacher delivering it and they've got no experience and it's almost like, well why are you telling me? You don't know anything about it.*

(Young People's Consultation Group 1)

Table 1, Table 2 show that programmes are seeking to involve young people themselves in the design and delivery of programmes. This was seen as advantageous by both young people and experts consulted and the UK grey literature yielded examples of this approach such as the ‘whole school’ model evaluated by Maxwell et al. (2010). This involved young people as researchers, as programme designers and in programme delivery. Members of the young people's group argued that such approaches assisted in investing programmes with authenticity and described information that featured or was delivered by young people themselves as more ‘real’ and ‘closer to home’. Experts from both consultation groups and the many of the international experts interviewed were similarly enthusiastic about the benefits of involving young people as ‘co-producers’ or peer mentors:

*…programmes that are able to use peers, students as part of the programme…I'm using role models for the students versus ‘here's an adult coming in and telling me about this stuff and what do they know, they don't know my life’.*

(Expert 1, Canada)

However, discussion in both the education and the communication and campaigning consultation groups emphasised the importance of peer mentors receiving relevant training and support.

#### 4.4.4 Responding to disclosures

We noted above the influence of high risk groups of participants on programme outcomes. The consultation with both young people and expert groups flagged up the issue of disclosure of IPVA which interventions might evoke. Evidence from the qualitative literature reviewed and members of the young people's consultation group argued the case for school-based interventions to be linked to appropriate services for those who disclosed experiences of abuse in their own or their parents' relationships:

*‘It makes people aware but then they need the help afterwards’*.

(Young People's Consultation Group 1)

Managing such disclosures was also identified as a potential source of concern for schools. The consultation identified differing views as to who should provide support following a disclosure in school of domestic abuse either in a young person's own intimate relationship or in their family. Whilst some of those contributing to the education consultation group considered school staff to be the appropriate people to receive and respond to such disclosures, others emphasised the need for more specialist forms of support which were located outside schools. Young people themselves emphasised the need for such support to be confidential and expressed doubts as to whether teachers could ensure this. They wanted young people to be informed about the availability of relevant support at an early stage and to be apprised about the consequences of disclosure:

*I think that they should bring to light what actually happens after you call the people…*.

(Young People's Consultation Group 1)

## 5 Discussion

IPVA prevention is a burgeoning field and we are aware that, since the end of the prespecified date range for this review, new papers have been published in this area: for example, a special issue of the Journal of Adolescent Health published in 2015 included a number of relevant studies. In line with standard systematic review methodology, these and any other papers that might be revealed by an updated systematic search will be included in the analysis when the review is updated in the future.

This review raises a number of questions about how preventive interventions in domestic abuse are theorised and evaluated. Most of the papers included in the systematic review failed to provide robust evidence of behaviour change, but using behaviour as a primary outcome of an intervention which is targeting social norms may be problematic. Many public health interventions aimed at whole populations aim to change behaviour by changing attitudes and knowledge, and shifts in attitudes and knowledge represent positive short-term and medium-term outcomes. Moreover, social norms are only one risk factor among a number of risk factors for IPVA.

The consultation arm of this study also found some key differences in respect of identifying intervention aims and measuring outcomes between the stakeholders involved in implementing these interventions. Most of these programmes originated in the IPVA practise, research or policy sectors, but schools are tasked with delivery. Whilst those participating in the consultation groups who worked in the domestic abuse sector were likely to identify the aims of programmes as the reduction of IPVA, education professionals were more focused on changing attitudes, arguing that children were exposed to a wide range of influences outside school and that changing behaviour was too ambitious an objective for education on its own. These education professionals had rather different ideas about what might constitute appropriate outcome measures for these interventions and suggested that measures of wellbeing or perhaps more tightly defined outcomes relating to help seeking, such as use of a helpline or knowledge of where to access help, should be utilised. These suggestions contrast with those of Fellmeth et al. (2013) who noted the lack of evidence regarding physical or mental health outcomes for young people participating in these interventions and suggested that more use be made of these measures.

The findings concerning the need for these programmes to be linked to services for those young people who disclose IPVA in their own or their parents' relationships exposes the overlap between primary prevention for whole populations and secondary prevention aimed at those who show early signs of experiencing IPVA (Wolfe & Jaffe, 1999). Targeting programmes on the general population of children and young people means that audiences will include those who have already experienced IPVA and the older the audience of children and young people, the more likely it is that they will have experienced IPVA in their own relationships. This, together with the perception that attitudes are more flexible and open to influence when children are younger, may indicate the importance of delivering these interventions earlier and examples of programmes designed for children under 10 were identified in the UK grey literature.

Any measurement of broader outcomes such as wellbeing or health would need to take account of secondary support services offered to young people identified as experiencing IPVA. At present, there are very few such services available in the UK and schools themselves often lack the expertise to take on this work of responding to disclosures. This is a key barrier to implementing these programmes more widely and was cited as a reason why schools may be reluctant to deliver these interventions. The other policy gap in England concerns the current lack of government support for making these programmes a mandatory part of the curriculum.

## 6 Conclusion

This review has identified some of the elements that contribute towards making programmes successful. Whilst off-the-shelf programmes are inevitably influential, there are strong arguments for including local elements in programme design and content and for ensuring that those who will be both delivering and receiving the intervention contribute to its development. The involvement of children and young people in the design and implementation of these interventions has the potential to increase their authenticity and this emerged as important to young people themselves. This involvement can be achieved by a variety of means including incorporating material co-produced with young people into programmes; through engaging them in participative learning activities such as drama and by training and involving them as peer mentors or facilitators. Organisational readiness was also identified as key and both evaluators and those planning programmes might consider employing a ‘maturity matrix’ to assess organisational readiness to implement.

We have aimed to draw attention to the context in which these programmes are delivered. If schools are to take on responsibility for implementing preventive interventions in domestic abuse, they require more preparation and fuller engagement in the task. At present, the delivery of these programmes can be apprehended as a role that has been imposed on them by other sectors. Incorporating domestic abuse prevention into national curricula, teacher training and school inspection would locate it more centrally in the education agenda. Measuring outcomes that emphasise the acquisition of learning and knowledge may also be more meaningful in the context of education.

## Figures and Tables

**Table:** 

Include	Exclude
Papers and reports published/dated between 1990 and 2012, updated to February 2014	Papers and reports published/dated before 1990
Published in any language	No language restrictions
Peer-reviewed research papers: all countries	Research papers that are not subject to peer review
Meta-analyses, research reviews, controlled studies, before-and-after studies, independent case evaluations, qualitative and ethnographic studies	In-house evaluations, internal audits
Qualitative studies that do not include the views of children and young people participating in interventions using their direct quotes
Children and young people at or below the age of 18	Studies with minimal or no data relevant to children/young adults below 18
Studies focused on prevention programmes for adults who perpetrate abuse
Studies including interventions to prevent domestic abuse	Studies focused only on child abuse and neglect or on bullying
Studies including children/young people in the general population	Studies only including children and young people who have experienced domestic abuse
Studies only including children and young people who have perpetrated domestic abuse
Studies of interventions aiming to prevent children and young people becoming either/both victims or perpetrators of domestic abuse	Studies focused only on prevalence or outcomes of domestic abuse

**Table:** 

Population	Intervention	Context	Outcome (general)	Outcome (specific)
Child* OR	Prevent* OR	Media OR	Outcome OR	Domestic AND ((abuse OR violen* OR batter*)) OR
Young person OR	Educat* OR	Communit* OR	Cost OR	Home AND ((abuse OR violen* OR batter*)) OR
Young adult OR	Train*OR	Public* OR	Cost analysis OR	Family AND ((abuse OR violen* OR batter*)) OR
Young people OR	Teach* OR	School*	Cost effectiveness OR	Families AND ((abuse OR violen* OR batter*)) OR
Adolescen* OR	Promot*OR	College	Acceptabl* OR	Gender AND ((abuse OR violen* OR batter*)) OR
Teenager* OR	Instruct*OR	School-based	Effective* OR	Spous* AND ((abuse OR violen* OR batter*)) OR
Youth*	Campaign* OR		Experience* OR	Partner* AND ((abuse OR violen* OR batter*)) OR
	Social Marketing OR		View* OR	Fiancé AND ((abuse OR violen* OR batter*)) OR
			Attitude* OR	Cohabitant*AND ((abuse OR violen* OR batter*)) OR
			Help seeking OR	Intimate AND ((abuse OR violen* OR batter*)) OR
			Protective Behaviour*OR	Interpersonal AND ((abuse OR violen* OR batter*))OR
			Harm reduction OR	Dat*AND ((abuse OR violen* OR batter*)) OR
			Healthy rel*OR	Relationship AND ((abuse OR violen* OR batter*)) OR
			Respectful rel*OR	Marital AND ((abuse OR violen* OR batter*)) OR
			Resources	Conjugal AND ((abuse OR violen* OR batter*))
				Perpat*
				Victim*

**Table 1:** Summary characteristics for included randomised studies.

Author date country	Programme title	Programme design	Study quality	Youth input?	Compliance and fidelity	Resource needs (high, med, low)	N youth included in final sample (intervention: control)	N sites	Context	Outcomes affected *(all small changes unless noted)*^a^, ^b^
Avery-Leaf et al., 1997 USA	No specific title	Five session curriculum	C/D (pilot)	N	Not reported	**M**	102 treatment/90 control 55% female overall: 63% of control group	1 (health classes randomised)	One school year. Grades 9–12. Almost 80% White. Lower middle class	Attitude (short term) Increased acceptance of aggression male/female and vice versa
Foshee et al., 1998 USA	Safe Dates	Ten sessions of 45 min	B	N	Y 90.7% of curriculum delivered	**H**	Total n 1700/1886 (n by group not given as analysis by school)	7:7	8th and 9th grade (13–15). High levels of dating violence at baseline (1:3).	Attitude, knowledge, incidence *(short term)* Large changes in knowledge scores
Foshee et al., 2000 USA	Safe Dates: 1 year follow up	1603 left in at 1 yr	7:7	As for code 64	Attitudes, knowledge *(longer term)*
Foshee et al., 2004 USA	Safe Dates + booster; 4 years	460	5:5	8th grade only: sub-randomised to booster or not	Incidence *(longest term)*
Foshee et al., 2005 USA	Safe Dates: 4 years individual analysis	1566 left in analysis (636 treatment/930 control: those who received the booster excluded: analysis by individual: those	7:7	As for code 54	Knowledge, Attitude, incidence *(longest term)* Moderate effects Incidence *(longest term)* Small effects, wide CI's
Foshee et al., 2012 USA	Families for Safe Dates	Leaflets sent out to parents × 6 (‘full treatment’ group)	B	N	Y : 88% of the treatment Families began the programme and 69% completed all six booklets.	**M**	1237 eligible households, 514 responses (37.1%). 140/230 in ‘full treatment’ arm completed follow up (61%) 184/234 control completed follow up (79%) 62% girls in treatment group vs 55% in control group 86% caregivers high school education in treatment vs 80% control. Other baseline demogs that are reported are similar	N/A	Families with teenagers	Knowledge, attitude, behaviour, incidence *(medium term)* *All small or moderately small effect sizes except caregiver acceptance of DV.*
Jaycox et al., 2006 USA	Break the Cycle	Three hours over 3 days of programme, run by lawyers who were activists in the area of DV	B	Y	On average 69% of curriculum covered	**M** (basic: only 3 h but with lawyers) **M** (additional activities)	1384/1941; 1156/1859	55:55	Latino/a population: US culture where legal solutions are the norm. All ages	Knowledge, attitudes, behaviour (*short term*) Knowledge Behaviour (*longer term*)
Miller et al., 2012 USA	Coaching Boys into Men	Coaches discuss 11 key messages in 10–15 min sessions over 12 weeks in sports training sessions	A/B	N	60% full compliance by coaches	**M**	847/1008: 951/998	8:8	US athletic culture. All ages	Knowledge, behaviour, incidence *(short term)*
Pacifici et al., 2001 USA	No specific title	Three 80 min sessions plus time to view a video	B	Y ‘videos to create credible communication through peers’	Not reported	**L**	/239:/219Tot: 458/547	2	Mainly 10th grade students	Attitude, only for subgroup more likely to be higher risk at baseline, and after data modelling *(short term)*
Taylor et al., 2013 USA	Shifting Boundaries	Classroom: Six sessions over 6–10 weeks. Building: creating building restraining orders, poster, hotspot mapping by students.	B	Y	Not reported	**M**	2655 in total: allocation between groups not specified	30 Group allocation not specified	6th 7th grade 40% of participants had been in prior violence prevention programmes. Very deprived communities. 85% non-white. More than half under national expected academic achievement	Incidence *(longer-term)* Reduction in DV in building only programme, but some non-DV risk behaviours increased
Taylor, Stein, & Burden, 2010a USA	No specific programme title	Five classroom periods 40 min each.	B	N	Not reported	**M**	1639 in total: allocation between groups not specified	123 classrooms: Group allocation not specified	6/7 grade. Wide ethnic mix	Knowledge, attitude, incidence *(short term)* Perpetration increased Knowledge, attitude *(longer term)*
Taylor, Stein, & Burden, 2010b USA	Gender differences in Taylor et al., 2010a	No gender effects
Wolfe et al., 2009 Canada	The 4th R	21 lesson curriculum: 28 h. Detailed lesson plans, videos,	B	Y (peer support as part of the programme)	Not reported	**M** (training for teachers) **H** (taught in 28 pre-existing sessions to both groups	754/916: 968/927	10:10	Grade 9 students	Incidence (*longer term*) at 2.5 yrs. reported DV 2.4% less but CIs for adjusted OR incl 1

a Short term = immediately after intervention and up to one month; medium term = up to 5 months after intervention; longer term = 6mths–under 4 years after the intervention; longest term = 4 or more yrs. after the intervention.

b All assessments based on at least p < 0.05 unless there are very large numbers of multiple tests when it is set at < 0.01.

**Table 2:** Summary characteristics for included non-randomised studies.

Lead author date country	Programme	Programme design	Study quality	Youth input?	Delivered with high compliance and fidelity	Resource needs (High, med, low)	N youth included in final sample (intervention: control)	N sites	Context	Outcomes affected (all small changes unless noted)*
Belknap et al., 2013 USA	Theatre intervention to prevent teen dating violence	School based: two plays (4 actors and the director) and a talkback session	B/C	?Y developing the plays highly iterative and based on prior qual work	Not reported	**M to develop** **L to deliver**	66	3 schools ? one class in each?	8th grade. High levels of poverty, 56% local community Latina/o. Most felt moderately unsafe locally	Attitude Behaviour *(short term)*
Bell & Stanley, 2006 UK	Healthy Relationships Programme		C for quant data B for qual data	N	Not stated	**M**	Cohort before and after (no control) — 55/85 completed final assessment	1 (1 class)	Year 8, one school, one class: high rates social exclusion, v low rates of academic success, marginalised community. Local DV services in place	Knowledge, attitudes, behaviour *(short term)* % change generally moderate
Black et al., 2012 USA	Dating Violence Prevention Project	Ten to twelve 50 min weekly sessions. Mix of same gender and mixed gender programmes (same gender all in one school; mixed gender all in the other school	C	Not noted	? Not noted, though biweekly meetings with facilitators intended to increase fidelity. 75–80% of those eligible participated	**M**	377/396 (intervention) 122/129 (control)	2	Very marginalised area, high absenteeism, low attainment, 99% African-Americans	Attitude *(short term)*
Elias-Lambert, Black, & Sharma, 2010 USA			B for satisfaction survey C/D for transfer of qual data to %							Girls more satisfied with programme than boys *(short term)*
Gardner & Boellaard, 2007 Canada	Connections: Relationship and Marriage	15 1 h sessions plus a student workbook. 4 units: personality (3 lessons), relationships 3 lessons), communication (2 lessons), marriage (7 lessons).	C/D	Not reported	Not reported	**H**	4 years post: 72/743 who did pre and post survey? (not clear how many completed the course): participants excluded if they took a further marriage course, and if couldn't be matched to a control	30 schools	Grades 11–12	Incidence *(longest term)*
Hilton et al., 1998 Canada	Antiviolence education	1 h fact giving assembly, then two 1 h workshops selected from 6 available.	C	N (but built on extensive testing of programme elements)	‘normal absentee rate of 10–20%’ for assembly. No other information	L	325/370/489 Based on 123/489 who did all three tests	4	Grade 11. Mixed urban/rural	Knowledge *(short term and medium term)* Some effect sizes moderate
Jaffe, Sudermann, Reitzel, & Killips, 1992 Canada	No specific programme	Range of different audio visual and external experts used (different content in different schools). Two schools half day, two schools full day	C/D (lower score due to incomplete reporting of data)	? possible – need to check prog design papers	? No data in this paper	**L/M**	627–629/737.	4	Low unemployment, relative affluence, mixed employment types, 90% + White	Attitudes *(short term)* Boys attitudes worse Attitudes *(medium term)* Attitudes worse
Katz et al., 2011 USA	Mentors in Violence Prevention	Ongoing iterative programme. MVP peer mentors/leaders chosen to closely mirror the ethnic and racial composition of the entire student body. N of mentoring sessions or other activities not stated.	A/B Well designed but no baseline data (only post-intervention comparison) so findings may be an artefact	Y	? not stated	**?M/H** **(not clear from text)**	894 (89%) intervention school 850 (91%) control school	2	Grade 9–12. approx. 50% White in both schools, but more Hispanic (23%) in Intervention school: 36% African American in control school	Attitude *(medium term)* Behaviour *(longest term)* Relatively large difference (largest mean difference 1.1/5)
Krajewski, Rybarik, Dosch, & Gilmore, 1996 USA	Skills for Violence-free relationships	Team teaching by teacher and battered women's counsellor of 10 consecutive health education class meetings (2 weeks).	B	Not apparent	Not stated	**M**	239 total — not clear how this divides between case and control	2	7th grade students 78.8% European American.	Knowledge Attitudes *(short term)* Attitude *(medium term)* Girls vs boys: more improvement
Lavoie et al., 1995 Canada	Prevention programme for violence in teen dating relationship	Short: Two classroom sessions (total 2–2.5 h). Long: 2 more sessions (added 2–2.5 h). Provided by one volunteer and one paid staff member from a community organisation	B/C	Not evident	No data	**L (short form)** **M (long form)**	Short: 279 Long: 238 57%/53% girls) Only those attending sessions and completing pre and post measures: not clear how they compare to population. Baseline scores better in short course school. May be systematic bias	2: one long form one short form	Inner city. French speaking. 10th grade. No other info	Attitude Knowledge *(short term)* Moderate differences in knowledge
Macgowan, 1997 USA	No specific title	Five 1 h sessions over 5 days. Developed by Domestic violence team. Presented by 5 teachers	C	Not evident	Y/N Fidelity assessed: Compliance not noted	M	247 girls (56%)193 boys (43.9% tot 440/802: 241 treatment/199 control more older and advanced level students in treatment group systematic exclusions applied	1	Grade 6–8. 72.3% black non-Hispanic. 8.3% White. No other data	Knowledge Attitudes *(short term)*
Miller et al., 2014 India	Coaching Boys into Men	Coaches discussed 12 key messages with male students who were cricket players in 45–60 min sessions over 4 months in sports training sessions	B	Y in prior qualitative work to develop the original programme for the Indian context	Y to an extent 80% of coaches completed all cards. 45% of participants reported exposure to 8–12 cards	**H**	663/741 completed baseline Q 309/663 completed follow up at 1 year (47%) — results only based on these 168/141 intervention/comparison	27/46 eligible: not clear why these and not the others	Age 10–16 Hindu and Muslim neighbourhoods. 2/3 in better-off housing, approx. 1/3 of mothers working Over 80% perpetrated violence at baseline.	Attitude *(longer-term)*
Wai Wan & Bateman, 2007 UK	No specific programme	Three 35 min sessions (constrained by national curriculum requirements). Mix of information giving, video, general and case-based discussion and small-group work	C	Not evident from description of design	? no data in this paper	**L/M**	100/107 intervention (58% female) 59/97 (47% female)	2 (one case one control)	No data given (though inner city schools NW England)	Knowledge Attitudes *(short term)*
Weisz & Black, 2001 USA	Reaching and Teaching Teens to Stop Violence	Twelve 1.5 h sessions. 2 co-trainers per course (from rape counselling centre.	D	? role play based on local groups' experience	Not stated	M	46/27/21 intervention; 20/0/9 : comparison, by time point	1 (2 classes)	99% African American very low income many who had failed at other schools	Knowledge *(longer term)*
Wolfe et al., 2009 Canada	Fourth R	21 lesson curriculum: 28 h	A/B	Y in designing the scenarios and as lead actors in the role-play	? No data given	Not relevant for this element (see code 52 for info re 4th R programme)	96 intervention 100 controls 56% female Intervention and control group participants similar 98 randomly sampled video tapes for detailed teacher ratings (intervention: 19 girls 28 boys tot 47. Control 32 girls 19 boys tot 51)	6 of 20 in RCT 3 per arm: chosen for convenience	Grade 9. Location demogs similar to all 20 schools in Wolfe RCT	Behavoiur *(medium term)* Moderate effects, especially in girls No difference in Incidence (perpetration) in longer term
